# IS A UNIVERSAL TERM FOR OLDER ADULTS CULTURALLY APPROPRIATE? CONSIDERING PREFERRED TERMS IN AFRICA

**DOI:** 10.1093/geroni/igae098.3276

**Published:** 2024-12-31

**Authors:** Margaret Adamek, Gifty Ashirifi, Dolapo Adeniji, Abraham Teshome

**Affiliations:** Indiana University Indianapolis, Indianapolis, Indiana, United States; Indiana University Indianapolis, Indianapolis, Indiana, United States; Indiana State University, Avon, Indiana, United States; Indiana University Indianapolis, Indianapolis, Indiana, United States

## Abstract

Terminology matters. For consistency’s sake, one may argue that aging scholars should use one universal term for older adults regardless of what part of the world they are studying. Others may argue that the terms used by scholars in the Global South to refer to older adults should not be dictated by scholars in the Global North. If the term “elder” is a term of respect for older adults in Sub-Saharan Africa, should African scholars writing about aging be required to use the preferred term of the Global North? Can a universal term for older adults be culturally appropriate globally? With such questions in mind, we conducted a mixed methods study of preferred terms for older adults. Respondents included 78 African scholars and practitioners who completed an online survey. For this study we analyzed responses of 13 scholars from 6 African nations who participated in online focus groups. Using descriptive narrative analysis, our results indicate a wide variety of preferred terms for older adults including elder (Ethiopia), elderly, senior citizen, seniors, pensioners, and golden agers (Malawi). Many terms for older adults exist in local dialects—some connoting respect for elders, others that are derogatory. Respondents indicated older Africans prefer terms that convey respect, honor, and endearment. In scholarly dissemination efforts, we recommend a balanced approach with a universal term such as older adults for international audiences and yet leeway for native scholars to use the term for older people (e.g., “elder”) that is a best fit for their culture.

